# Safety Evaluation of Engineered Nanomaterials for Health Risk Assessment: An Experimental Tiered Testing Approach Using Pristine and Functionalized Carbon Nanotubes

**DOI:** 10.1155/2013/825427

**Published:** 2013-04-17

**Authors:** Teresa Coccini, Luigi Manzo, Elisa Roda

**Affiliations:** Laboratory of Clinical Toxicology, IRCCS Maugeri Foundation, Medical Institute of Pavia, and University of Pavia, Via Maugeri, 1027100 Pavia, Italy

## Abstract

Increasing application of engineered nanomaterials within occupational, environmental, and consumer settings has raised the levels of public concern regarding possible adverse effects on human health. We applied a tiered testing strategy including (i) a first *in vitro* stage to investigate general toxicity endpoints, followed by (ii) a focused *in vivo* experiment. Cytotoxicity of laboratory-made functionalized multiwalled carbon nanotubes (CNTs) (i.e., MW-COOH and MW-NH2), compared to pristine MWCNTs, carbon black, and silica, has been assessed in human A549 pneumocytes by MTT assay and calcein/propidium iodide (PI) staining. Purity and physicochemical properties of the test nanomaterials were also determined. Subsequently, pulmonary toxic effects were assessed in rats, 16 days after MWCNTs i.t. administration (1 mg/kg b.w.), investigating lung histopathology and monitoring several markers of lung toxicity, inflammation, and fibrosis. *In vitro* data: calcein/PI test indicated no cell viability loss after all CNTs treatment; MTT assay showed false positive cytotoxic response, occurring not dose dependently at exceedingly low CNT concentrations (1 **μ**g/mL). *In vivo* results demonstrated a general pulmonary toxicity coupled with inflammatory response, without overt signs of fibrosis and granuloma formation, irrespective of nanotube functionalization. This multitiered approach contributed to clarifying the CNT toxicity mechanisms improving the overall understanding of the possible adverse outcomes resulting from CNT exposure.

## 1. Introduction

Nanotechnology is one of the fastest emerging fields involving development and manipulation of materials ≤100 nm in size. There are numerous potential perspectives for the applications arising from nanotechnology which include their use in a wide range of fields, for example, medicine, environment, occupational setting, chemistry, energy production, information and communication, heavy industry, and consumer goods. Human exposure to engineered nanomaterials (ENMs) can occur at different stages of their life cycle (manufacture, use, and disposal) and several concerns have been expressed about their potential to cause unanticipated adverse effects in humans.

The remarkable diversity of engineered nanomaterials (ENMs), together with their unique properties and behaviour, complicates their risk assessment; there are currently about 50,000 different types of carbon nanotubes obtained by different raw materials and production processes. Similar diversity applies to other types of ENMs, rendering ad hoc risk assessment of all of these materials an immense task [1, 2].

The REACH Regulation (Registration, Evaluation, Authorization, and Restriction of Chemicals) is the current regulatory framework for chemical risk assessment and management in European Union. Although REACH should be applied to ENMs, the Technical Guidance Documents [3] for preparing a risk assessment currently include very little reference to substances in particulate form, thus lacking in addressing specific characteristics of ENMs. In 2007 and 2009, the European Union (EU) Scientific Committee on Emerging and Newly Identified Health Risks (SCENIHR) concluded the applicability of the current risk assessment approaches/methodologies to identify the ENM-associated hazard, though pointing to main limitations, thus stressing the need for several adjustments [4–6].

Nevertheless, though particular ENMs properties and behaviour (i.e., translocation and capability to cross biological barriers) have been described in several studies (i.e., mode of action, toxicity targets, dose-response relationships, and the potential to react with constituents of cells at the portal of entry and beyond [2]), several aspects concerning NP-associated risks are still unknown, and critical steps in the risk assessment of ENMs remain so far the same as those used for conventional chemicals. 

Furthermore, there are major challenges in assessing exposure (both airborne/administered and internal dose, i.e., particle deposition in lung). ENMs are produced from many substances, in many forms and sizes, and with a variety of surface coatings. The health risk assessment for such diverse materials requires validated analytical methods permitting their characterization in bulk samples and their detection and measurement in workplace air [7]. Efforts are also needed for the identification of properties that trigger ENMs toxicity (e.g., contaminants, impurities, and defects). 

Today, there are not enough studies to validate the potential hazards posed by these novel materials and hence the definitive conclusions and tools, technologies, systems, and methods to obviate the risks. Several tools for the assessment of risks are still in the conceptual stage, and further, there are considerable uncertainties on how to assess nanoparticles (NPs) exposure (i.e., which metrics to adopt systematically) and what methods to use to assess toxicity.

Thus, being unable to perform a quantitative risk assessment for ENMs, due to the lack of sufficient data on exposure, biokinetics, and organ toxicity, it should be made mandatory to prevent exposure by appropriate precautionary measures and practicing best industrial hygiene to avoid future shock scenarios from environmental or occupational exposures [8].

In the safety assessment area, two basic questions still need to be addressed: (i) do nanomaterial properties necessitate a new toxicological science? and (ii) what are the biological and biokinetic properties to be considered for toxicity testing; in particular, can biology and certain mechanisms of effects of ENMs (e.g., proinflammatory action, oxidative stress, mitochondrial perturbation, generation of neoantigens and protein complexes in the body, enhanced protein degradation at the large surface area of NPs complexes, etc.) be used as a basis for studying NPs toxicity and risk assessment? Addressing these questions is of crucial importance to define adequate strategies and establish whether ENM-tailored testing methods should be added to conventional toxicity testing protocols to comply with regulatory demand and properly characterize the ENMs potential hazard.

According to the major institutions [7, 9, 10] and international consensus meetings [11], the proposed multitiered testing protocols could be used to address toxicological research and health risk assessment for NPs. Toxicity testing should firstly include a careful* physicochemical characterization *(e.g., particle size, particle size distribution, reactivity, surface area, particle mass, impurity, and aggregation tendency), assisted by using reference materials, as well as the use of acellular systems to explore the reactivity of the materials in acellular environments. Subsequent steps include the use of *validated cellular (in vitro) models* to support evidence-based testing process, followed by* a limited series of in vivo studies *guided by information generated from *in vitro* studies, especially the toxicological data relevant to ranking of ENMs, designing appropriate exposure concentrations and defining the critical health endpoints to be monitored.

Biosafety should be evaluated by tests examining general toxicity, target organ toxicity, and biocompatibility in line with regulatory requirements, limiting the use of lab animals in toxicological research [12, 13], to identify molecular endpoints and multiple toxicity pathways. 

The present work represents an example of a tiered approach for toxicity testing, employing CNTs as a practical model suitable to validate a two-level strategy combining *in vitro* and *in vivo* experiments. Such investigation is related to our recent studies on the pulmonary toxicity of a series of functionalized multiwalled carbon nanotubes (MWCNTs).

Human A549 pneumocytes, selected as potential target cells during respiratory exposure associated with occupational setting and environment [1, 7, 14–18], have been used to assess *in vitro* the cytotoxicity of MWCNTs, with different degrees of functionalization, using MTT assay and calcein/propidium iodide (PI) staining. 

Subsequently, the pulmonary toxic effects were assessed in rats 16 days after i.t. administration of the previously reported type of CNTs (1 mg/kg b.w.). Major endpoints tested included (i) histopathology of lung tissue (haematoxylin/eosin staining), (ii) apoptotic/proliferating features examined by TUNEL and PCNA immunostaining, and (iii) presence/distribution of (1) transforming growth factor-beta1 (TGF*β*1), (2) interleukin-6 (IL-6), and (3) collagen (Type I) investigated by immunochemical methods, as markers of lung toxicity, inflammation, and fibrosis, respectively.

### 1.1. CNTs: State of the Art

Among the several types of engineered nanoparticles, CNTs are emerging as one of the most promising and revolutionary nanomaterials, widely employed within commercial environmental and energetic sectors, due to their unusual one-dimensional hollow nanostructure and unique physicochemical properties [19, 20]. CNTs have been also proposed in medicine as nanovectors for vaccine and drug delivery, nanodevices, or as substrates for tissue engineering [21, 22]. Consequently, this expanding usage, paralleled by occupational exposure at all phases of the material life cycle, may lead to widespread human exposure via skin contact, inhalation, and via intravenous injection in medical applications.

Moreover, in view of some similar aspects to fibers, such as structural characteristics, extreme aspect ratio, low specific density, and low solubility, CNTs might exhibit toxicity similar to those observed with other fibrous particles such as asbestos [1]. Further, their small size accompanied by high surface area defines the chemical reactivity of CNTs inducing changes in permeability and conductivity of biological membranes; in fact, a typical behaviour has been reported regarding translocation and distribution of certain ENMs in the body and their capability to cross internal barriers [2]. Moreover, chemical functionalization not only extends the applications of CNTs conferring them new functions that cannot otherwise be acquired by pristine nanomaterials, but also impacts on biological response to CNTs modifying their toxicological profile [22, 23].

Thus, the growing use together with the suspense whether CNTs have negative impact on human health and environment evokes concern by worldwide public and regulatory institutions. 

Nowadays, a number of *in vivo* and *in vitro* studies have also been performed on CNT toxic effects, evaluating different mechanistic endpoints [14, 24–30]. However, the existing data are controversial and findings have been difficult to be interpreted in some cases. Toxicity and reactivity of CNTs were shown to vary depending on characteristics of the specific type of material tested, such as fiber length, fiber diameter, surface area, tendency to agglomerate, dispersibility in media, and the method used for synthesis which can produce impurities and leave metal catalyst residues [31–38].

Some scientists have suggested that metal traces associated with the commercial nanotubes are responsible for the cytotoxicity and that CNTs show no signs of acute toxicity [29]. It has also to be taken into consideration that *pristine* CNTs are insoluble in almost all solvents, and, effects of CNTs can also be modified by chemical functionalization [39].

In certain studies, a high degree of functionalization was shown to mitigate toxic effects [39, 40], while, contrarily, in other studies, functionalization increased lung toxicity of CNTs in mice exposed by inhalation [41]. Further, dissimilar results from *in vivo* studies have been ascribed to differences in CNT size and surface area, rodent species used, route of exposure, and differences in observation period [42–47].

## 2. Materials and Methods 

### 2.1. Raw Material

Commercial MWCNTs (95% purity) were obtained from Cheap Tubes Inc. (Brattleboro, USA) and CB from Aldrich-Fluka (Milan, Italy). SiO_2_ particles were kindly provided by Degussa AG (Germany) (Table 1).

### 2.2. Preparation of Lab-Made Functionalized MWCNTs

MWCNTs with different functionalization were prepared as described by Fagnoni et al., 2009 [48]. The lab-made materials produced included carboxyl (COOH) functionalized MW-COOH and amine-containing nanotubes with low amino groups content (MW-NH_2_). 

#### 2.2.1. Physicochemical Characterization of CNTs

Before the *in vitro* and *in vivo* exposure, exhaustive physicochemical characterization of differently functionalized CNTs was performed by IR spectroscopy, thermogravimetric analysis (TGA), and by noncontact atomic force microscopy (NC-AFM).

It has been estimated that one functional chain is anchored each ~16 carbon atoms of the CNTs surface for the MW-COOH and each ~100 carbon atoms of the CNTs surface for the MW-NH_2_.

All the three types of CNTs had an outer diameter of 20–30 nm and the wall thickness was 1-2 nm. The particle length was 100–300 nm for both functionalized CNTs (e.g., MW-COOH and MW-NH_2_) and 500–2000 nm for the pristine MWCNTs. Other physicochemical characteristics are detailed in Table 1.

The content of iron (Fe), cobalt (Co), and nickel (Ni) in both *pristine* and functionalized MWCNTs was determined by inductively coupled plasma optical emission spectrometry (ICP-OES) analysis. Results indicated the presence of Fe and Ni impurities at concentrations of 0.25% and 1.5%, respectively, in *pristine* MWCNTs. Considerably lower metal concentrations were found in the functionalized nanotubes. In these materials, the content of Fe and Ni was 0.05%–0.15% and 0.3%–0.5%, respectively.

#### 2.2.2. Nanotubes Dispersion

Previous to the experiments, a stock of 1 mg/mL and a suspension of 1 mg/kg b.w. of each test material (i.e., MWCNT, MW-COOH, MW-NH_2_, SiO_2_, and CB) were prepared in DMEM plain or in NaCl 0.9% for the *in vitro* expo pure and *in vivo* exposure, respectively. Just before use, these suspensions were sonicated for 15 min with an ultrasonic Sonopuls (Bandelin Electronics, Berlin, Germany) in a short break every two minutes, also vortexing the suspension on ice to further force the CNT dispersion, avoiding the agglomeration and the formation of bundle-like structures. No surfactants or solvents were used. The suspensions of the test materials were immediately used for the treatment. 

### 2.3. *In Vitro* Tests

#### 2.3.1. A549 Human Cells

All cell culture reagents were obtained from Sigma Aldrich (Milan, Italy). A549 cells from a human Caucasian lung adenocarcinoma with the alveolar type II phenotype were obtained from ECACC (Sigma Aldrich, Milan, Italy). The cells were cultured in Dulbecco's modified Eagle medium (DMEM) supplemented with 10% fetal calf serum (FCS), 2 mM L-glutamine, 50 IU/mL penicillin, and 50 *μ*g/mL streptomycin, in a humidified atmosphere containing 5% CO_2_ at 37°C and grown to 80% confluence. Exposures to the different nanomaterials were done on subconfluent cells. 

At the end of the incubation period, cell cultures were examined by phase contrast microscopy using a Zeiss Axiovert 25 light microscope.

#### 2.3.2. Exposure Conditions

Cells were seeded in 96-well plates at a density of 1  × 10^4^/cm^2^ in complete medium. After 24 h of cell attachment, plates were washed with 100 *μ*L/well of phosphate-buffered saline (PBS). Cells were then exposed to suspensions (10 *μ*L) of the test materials (pristine MW-CNTs, MW-COOH, MW-NH_2_, CB, or SiO_2_) at concentrations as 1, 10, and 100 *μ*g/mL, for 24 or 48 h. No fetal bovine serum was used in these preparations as it was proven to interact with nanotubes [49].

The doses tested (1–100 *μ*g/mL) and the time points of determinations (24 and 48 hr) have been derived from previous studies showing different adverse effects (e.g., oxidative stress, inflammatory response, cell death, loss of cellular morphology, and gene expression levels changes) caused by various carbon nanotubes.

Six replicate wells were used on each 96-well plate for all the treatments, and each experiment was repeated independently three times. Identical treated cultures were taken as replicate measure for statistical tests. Significant effects (*P* < 0.05) were determined by one-way analyses of variance (ANOVA) (software package SPSS Inc. 1999).

In graph figures, data are presented as mean ±  SD over the mean experimental values of each of the three independent experiments. 

#### 2.3.3. Cytotoxicity Assays

Two dye-based methods, namely, the Live (calcein-green fluorescence)-Dead (propidium iodide-red fluorescence) staining (calcein/PI) and the MTT assay were used to assess cytotoxicity. 

The live/dead viability test detects cell membrane integrity and measures the number of damaged cells. Fluorescence microscopy labels both live (green colour) and dead (red colour) cells simultaneously thereby permitting visualization and enumeration of all cells by a single counting procedure. Viability was expressed as a percent of the total number of cells counted.

The MTT assay uses tetrazolium salts to assess mitochondrial dehydrogenase activity. Only active mitochondria contain dehydrogenases enzymes able to cleave the tetrazolium ring and reduce MTT to insoluble dark-blue formazan crystals and, therefore, the reaction only occurs in viable cells. 

Absorbance, directly proportional to cell viability, was determined at 550 nm in a Biorad microplate reader. The absorbance values were normalized by the controls and expressed as percent viability.

After the incubation period, all cultures were also examined and photographed in phase contrast using a Zeiss Axiovert 25 light microscope combined with a digital camera (Canon Powershot G8).

### 2.4. *In Vivo* Tests

#### 2.4.1. Animals and Treatment with Carbon Nanotubes by Intratracheal Instillation (i.t.)

All experimental procedures involving animals were performed in compliance with the European Council Directive 86/609/EEC on the care and use of laboratory animals.

Adult Sprague-Dawley rats (25 males, 12 weeks old) were purchased from Charles River Italia (Calco, Italy) at least 2 weeks before treatment and allowed to acclimatize for 3 weeks. Throughout the experiment, animals were kept in an artificial 12 h light : 12 h dark cycle with humidity at 50 ± 10%. Animals were provided with rat chow (4RF21 diet) and tap water ad libitum.

For the treatment, groups of 6 rats were anesthetized with pentobarbital sodium for veterinary use and were intratracheally instilled with MWCNTs, pristine or functionalized, dispersed at a dose of 1 mg/kg b.w. in NaCl 0.9% (see Section 2.2.2). 

Sixteen days after the i.t., treated and control rats were deeply anesthetized with an overdose i.p. injection of 35% chloral hydrate (100 *μ*L/100 g b.w.); lung preparation for microscopic evaluations was done by vascular perfusion of fixative. Briefly, the trachea was cannulated, and laparotomy was performed. The pulmonary artery was cannulated via the ventricle, and an outflow cannula was inserted into the left atrium. In quick succession, the tracheal cannula was connected to about 7 cm H_2_O pressure source to inflate the lungs with air, and clearing solution (saline with 100 U/mL heparin, 350 mosM sucrose) was perfused via the pulmonary artery. After blood was cleared from the lungs, the perfusate was switched to fixative consisting of 4% paraformaldehyde in 0.1 M phosphate buffer (pH 7.4). After fixation, the lungs were carefully removed, washed in NaCl 0.9%, and postfixed by immersion for 7 h in 4% paraformaldehyde in 0.1 M phosphate buffer (pH 7.4); then, the tissues were dehydrated through a graded series of ethanol and finally embedded in Paraplast. Eight *μ*M thick sections of the samples were cut in the transversal plane and collected on silane-coated slides.

#### 2.4.2. Histology, Immunocytochemistry, and TUNEL Staining

To avoid possible staining differences due to small changes in the procedure, all the reactions were carried out simultaneously on slides of control and treated animals at all stages. 

Lung sections were stained with haematoxylin/eosin (H&E) for histological examination.

Immunohistochemistry was performed using commercial antibodies on rat lung specimens, to assess the presence and distribution of (i) transforming growth factor-beta1 (TGF*β*1), (ii) interleukin-6 (IL-6), (iii) collagen (type I), and (iv) proliferating cell nuclear antigen (PCNA-PC10), as typical markers of general lung toxicity, inflammation, fibrosis, and cell proliferation, respectively.

Lung sections were incubated overnight at room temperature with (i) a primary rabbit polyclonal antibody against TGF*β*1 (Santa Cruz Biotechnology, Santa Cruz, CA, USA) diluted 1 : 100, (ii) a primary rabbit polyclonal antibody against collagen (type I) (Chemicon, Temecula, CA, USA) diluted 1 : 400, (iii) a primary goat polyclonal antibody against IL-6 (Santa Cruz Biotechnology) diluted 1 : 100, (iv) a primary mouse monoclonal antibody against PCNA (American Biotechnology, Plantation, USA) diluted 1 : 5 in PBS. Biotinylated anti-rabbit, anti-goat, and anti-mouse secondary antibodies and an avidin biotinylated horseradish peroxidase complex (Vector Laboratories, Burlingame, CA, USA) were used to reveal the sites of antigen/antibody interaction. 3,3′-Diaminobenzidine tetrahydrochloride (DAB; Sigma, St. Louis, MO, USA) was used as the chromogen, followed by a light counterstaining with haematoxylin. Then, the sections were dehydrated in ethanol, cleared in xylene, and finally mounted in Eukitt (Kindler, Freiburg, Germany).

In the case of negative controls, some sections were incubated with phosphate-buffered saline instead of the primary antibodies; no immunoreactivity was observed in these conditions.

In addition to morphological criteria, the apoptotic cell death was assayed by *in situ* detection of DNA fragmentation using the terminal deoxynucleotidyl-transferase (TUNEL) assay (Oncogene Res. Prod., Boston, MA, USA). The lung sections were incubated for 5 min with 20 *μ*g mL^−1^ proteinase-K solution at room temperature, followed by treatment with 3% H_2_O_2_ to quench endogenous peroxidase activity. After incubation with the TUNEL solution (90 min with TdT/biotinylated dNTP and 30 min with HRP-conjugate streptavidin) in a humidified chamber at 37°C, the reaction was developed using 0.05% 3-amino-9-ethylcarbazole (AEC) in 0.1 M TRIS buffer (pH 7.6) with 0.2% H_2_O_2_; in some specimens the reaction was developed using a 0.1% DAB solution. 

As a negative control, the TdT incubation was omitted; no staining was observed in these conditions.

#### 2.4.3. Cytochemical Assessment

 (a) Scoring different specimens, the immunostaining for IL-6, TGF*β*1, and collagen (*type I*) was evaluated in conventional brightfield microscopy by recording the localization and intensity of labelling according to a semiquantitative scale from absent/undetectable (−) to maximal (++++). Then, to assess the significance of the immunohistochemical results, a Kruskal-Wallis nonparametric analysis of the semiquantitative data was performed. A *P* value of <0.05 was considered significant.

(b) The evaluation of PCNA- and TUNEL-cytochemically positive cells (PCNA L.I., TUNEL L.I.) was calculated as the percentage (Labelling Index) of a total number (about 500) of bronchiolar, alveolar, and macrophagic cells, for each animal and experimental condition, in different representative microscopic fields. Statistical analyses among the different biological situations were performed by the Student's *t-*test, and differences between medians were considered significant at *P < 0.05.

The slides were observed and scored with a brightfield Zeiss Axioscop Plus Microscope. The images were recorded with an Olympus Camedia C-2000 Z digital camera and stored on a PC running Olympus software.

## 3. Results and Discussion

Laboratory-made functionalized multiwalled CNTs (MW-COOH and MW-NH_2_) were tested in comparison with *pristine* MWCNTs in cultures of A549 pneumocytes. Carbon black (CB) and silica (SiO_2_) were also investigated as reference nanoparticles [50, 51]. Purity and physicochemical properties of the tested nanomaterials are reported in Table 1.

*In vitro* cytotoxicity was assayed in parallel by two classical dye-based cell viability assays, for example, MTT metabolism and live/dead-calcein/PI staining.

**Figure 1:**
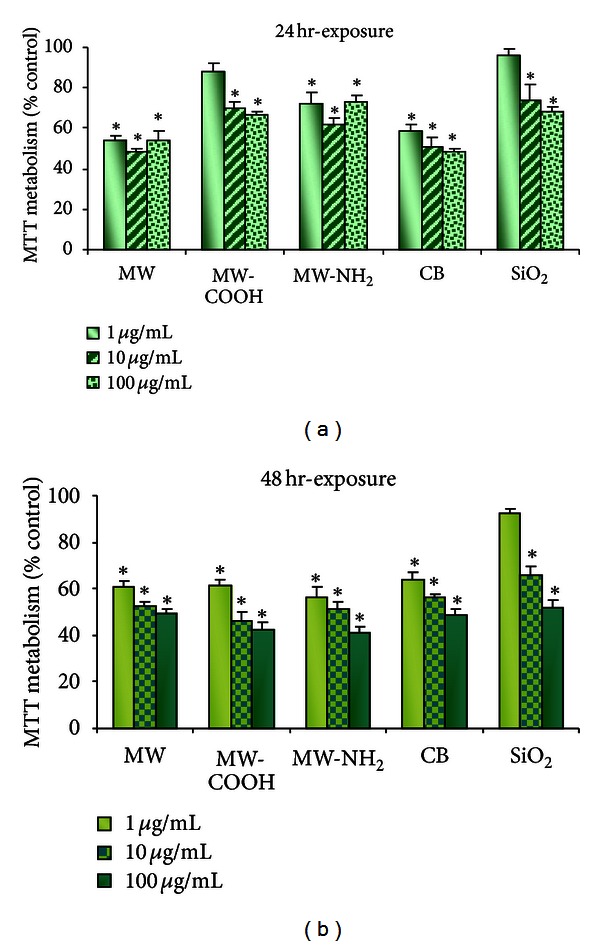
Histograms showing cell viability measured by MTT assay in A549 cells after 24 h and 48 h exposure to *pristine* multiwalled CNTs: MWCNT; laboratory-made functionalized nanotubes: MW-COOH (carboxyl functionalized) and MW-NH_2_ (amino-functionalized); carbon black: CB; silica: SiO_2_. Data represent the mean ±  SD over the mean experimental values of each of the three independent experiments.*P < 0.05 compared to control (100% cell viability).

**Figure 2:**
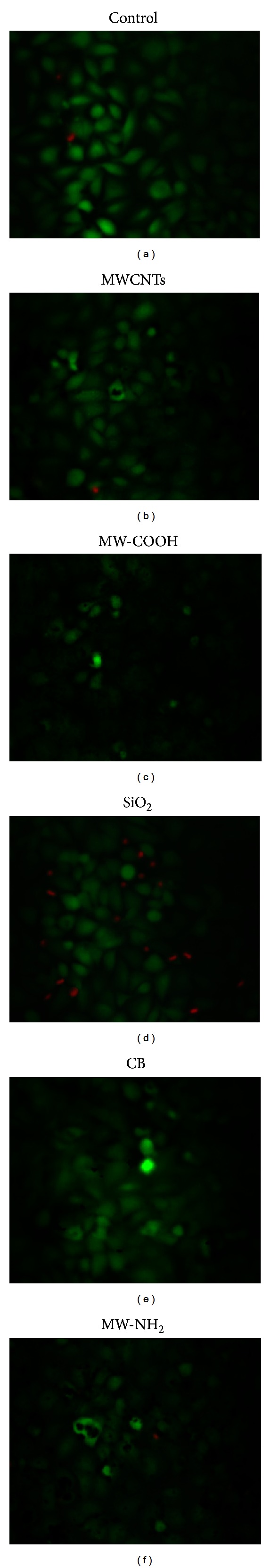
A549 cells examined by calcein/PI staining. (a) Untreated cells. Representative micrographs showing cell preparations after 24 h exposure to 100 *μ*g/mL: (b) *pristine* MWCNT; (c) MW-COOH: laboratory-made carboxyl functionalized nanotubes; (d) SiO_2_: silica; (e) CB: carbon black; (f) MW-NH_2_: laboratory-made amino-containing functionalized nanotubes. Viable (green) and damaged (red) cells are shown. *Objective Magnification: *32x (×4).

In preparations treated with all nanotube types (pristine and functionalized) and CB the calcein/PI test indicated no loss of cell viability (Figure 2), whereas MTT data of pristine MWCNTs, MW-COOH, and MW-NH_2_ apparently showed cytotoxic response, occurring not dose-dependently at exceedingly low CNT concentrations (1 *μ*g/mL), with 50% viability loss at 10 *μ*g/mL and no further cell death increase at 100 *μ*g/mL (Figure 1). Similar cytotoxicity pattern was observed by MTT in preparations exposed to CB. Loss of cell viability was apparently more pronounced with CB compared to SiO_2_ (Figure 1).

Notably, as shown in Figure 2, the data obtained by calcein/PI staining considerably differed from the MTT results in that (i) cell viability was unaffected after treatment with pristine MWCNTs, functionalized MWCNTs, or CB, even at the higher concentrations and for longer time of exposure (i.e. 48 h); (ii) loss of cell viability was noted in cells treated with SiO_2_ with approximately 40% cell death occurring after 24 h exposure to 100 *μ*g/mL SiO_2_ (Figure 2).

**Figure 3:**
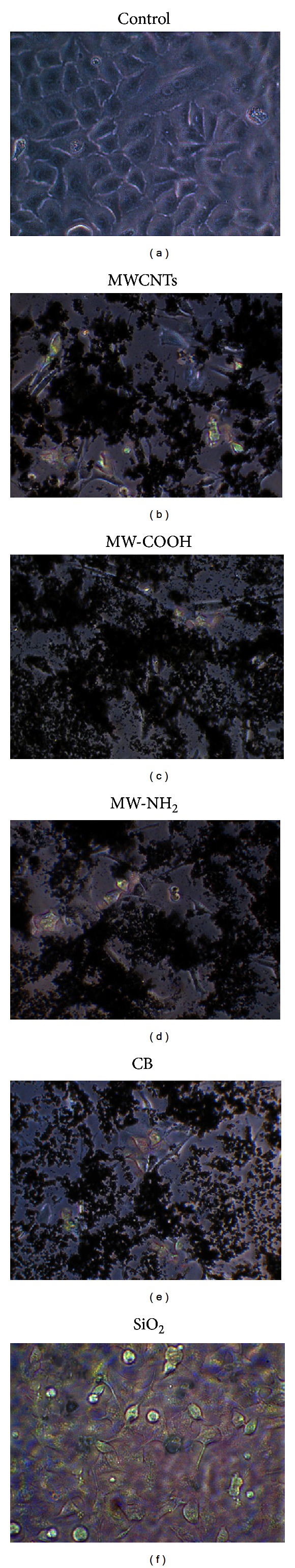
Phase contrast micrographs showing A549 cell cultures. (a) Untreated cells. (b–f) Bundle-like agglomerates covering cell surface after 24 h exposure to 100 *μ*g/mL. (b) MWCNTs, (c) MW-COOH, (d) MW-NH_2_, or (e) CB; (f) absence of agglomeration after exposure to SiO_2_ (100 *μ*g/mL). *Objective magnification: *32x (×4).

Pristine MWCNTs, MW-COOH, and MW-NH_2_ were either insoluble or little soluble in polar solvents and difficult to evenly disperse in an aqueous matrix. They were sonicated to force their dispersion in the medium before application to cell cultures. In cells incubated with these materials significant agglomeration occurred. CNTs were shown to adhere to each other forming dense micron-sized assemblies completely covering the cell surface (Figure 3). Agglomeration increased with increasing doses, at both incubation time points (24 and 48 h). The dosimetry of these nanoparticles tested *in vitro* is a fundamental question, including not only amount and time but also primary particle characteristics: physical properties (e.g., size, shape, and agglomeration state), core particle, and surface chemistry. The present data on (i) dose metric characteristics (Table 1), (ii) agglomeration tendency (Figure 3), and (iii) *in vitro* cytotoxicity suggest some considerations. Specifically, overt *in vitro* toxic effects were observed after exposure to SiO_2_ (viability results from both MTT and calcein/PI), while contrasting results were obtained from MTT and calcein/PI after pristine and functionalized MWCNTs. As far as physicochemical properties concern, although the functionalized nanomaterials possessed lower aggregate size and particle size ranges (Table 1) than those displayed by the pristine ones, these characteristics seemed not to markedly modify their cytotoxic potential when compared to that of pristine MWCNTs. 

Further, according to the literature, reporting interactions between CNTs and colorimetric dyes commonly used in classical dye-based cytotoxicity assays, such as MTT and neutral red assays [28, 51–54], the present MTT data seemed more likely to reflect a false positive cytotoxicity signal possibly due to nonspecific CNT interaction with cell culture components or MTT formazan salt. 

Subsequently, the second focused *in vivo* step addressed the pulmonary effects of the three different previously mentioned MWCNTs (*pristine* versus two diverse lab-made functionalized MWCNTs, i.e., MW-COOH and MW-NH_2_) on some cell kinetic (i.e., TUNEL and PCNA) and cytochemical parameters (i.e., IL-6, TGF*β*1, and collagen) investigated in rats after 16 days from a single i.t. exposure (1 mg/kg b.w.).

**Figure 4:**
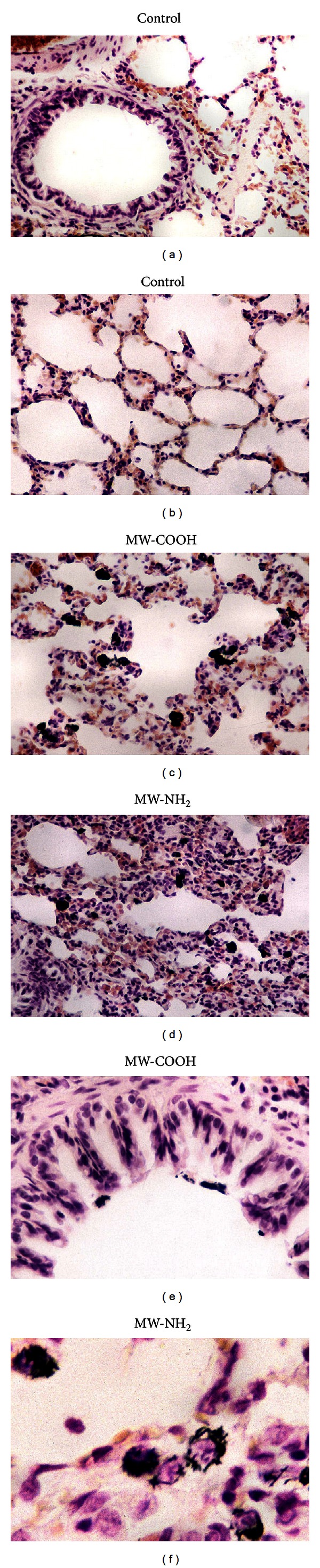
Representative H&E-stained lung parenchyma specimens. (a, b) Normal lung architecture in control animals. (c–f) Structural alterations detected in treated rats, i.t. exposed to 1 mg/kg b.w. MW-COOH (c, e) or MW-NH_2_ (d, f). Dark, particulate-laden macrophages, evident as a consequence of the carbon nanotubes engulfing, at alveolar (c, d), bronchiolar (e), and stromal (f) levels. (d) Thickened-walled collapsed alveoli accompanied by the presence of widespread microhaemorrhagic foci. *Objective magnification: *40x (a–d); 60x (e, f).

After i.t. exposure to all the three different CNTs, lung morphology micrographs clearly showed a marked uptake of the CNTs into the macrophages (Figure 4). Noticeably, the widespread presence of dark, particulate-laden macrophages was evident as a consequence of the CNTs engulfing at alveolar, stromal, and also bronchiolar levels in accordance with the poor solubility of the MWCNTs (*pristine* and lab-made MW-COOH and MW-NH_2_) already evidenced in the first *in vitro* step, as described previously and irrespectively of the nature of functionalization. 

Histologically, alteration of lung architecture was also observed in several areas showing collapsed thick-walled alveoli and presence of microhaemorrhagic foci (Figure 4), not accompanied by evident signs of fibrotic reaction. Occasionally, inflammatory shedding of leucocyte clusters characterized the parenchyma, even though the occurrence of true granulomas was never detected.

**Figure 5:**
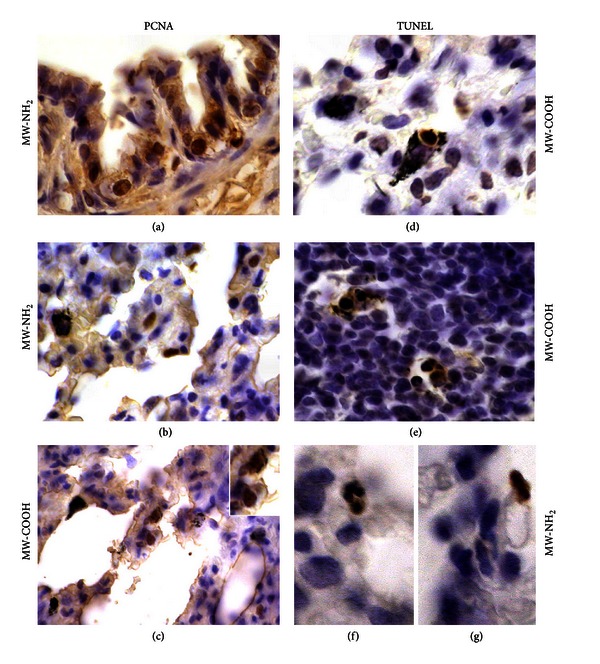
(a–c) Cell proliferation and (d–g) apoptosis, detected by PCNA immunolabelling and TUNEL staining, respectively, after *in vivo* exposure to 1 mg/kg b.w.: (c, d, e) MW-COOH and (a, b, f, g) MW-NH_2_. PCNA-positive epithelial cells at (a) bronchiolar and (b, c) alveolar levels; TUNEL-positive CNT-laden macrophages in both (d) normal and (e) inflammatory stromal areas as well as (f, g) at alveolar levels. *Objective magnification: *60x (a–e), 100x (f, g).

**Figure 6:**
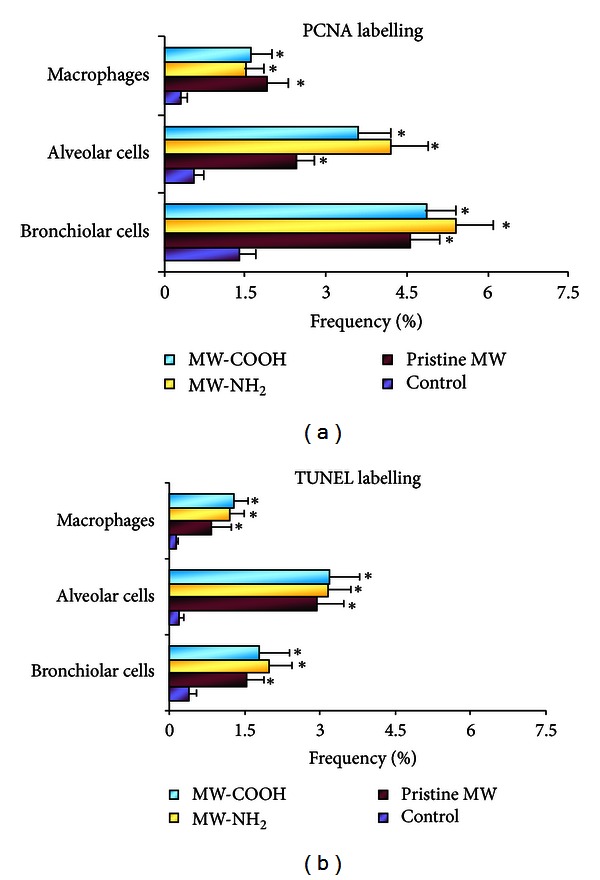
Histograms showing changes in percentage of (a) PCNA and (b) TUNEL Labelling Index of bronchiolar, alveolar and macrophagic cells after i.t. exposure to different CNTs (pristine MWCNTs versus lab-made functionalized MW-COOH and MW-NH_2_). A significant increase (Student's *t*-test) of the above-mentioned cell types was clearly observed in lungs from all CNT-treated rats. Data are expressed as mean ± S.D. (*P < 0.05).

TUNEL and PCNA staining, employed as typical markers of apoptosis and cell proliferation, respectively, showed a significant increase of reactivity in different cell populations (i.e., bronchiolar, alveolar cells and macrophages), as expression of (i) diverse sensitivity of different cell categories to the insult and (ii) an improved cellular turnover aimed at replacing damaged elements and finalizing a repair process (Figures 5 and 6).

**Figure 7:**
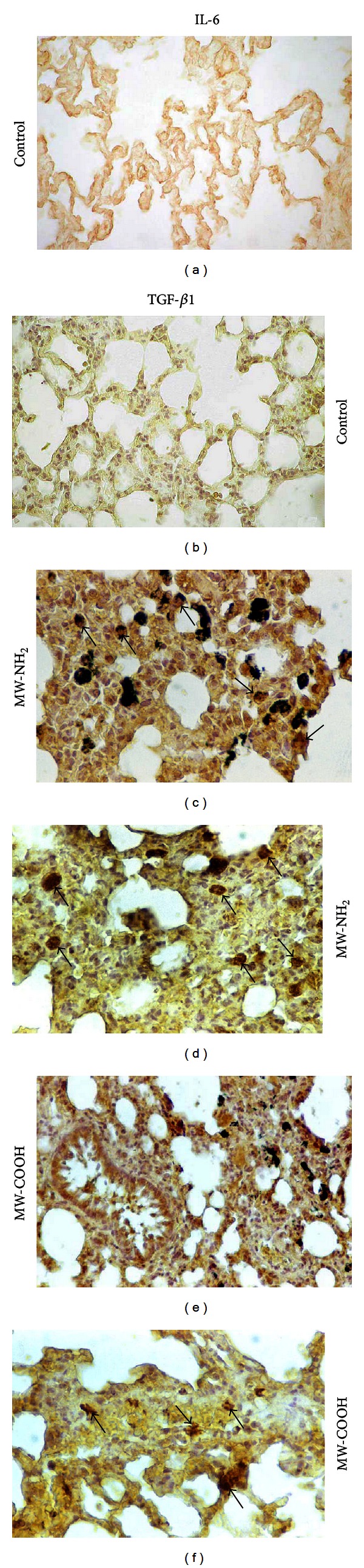
(a–d) *IL-6* and (e, f) *TGF-*β*1* immunostaining patterns in controls (a, b) and differently treated rats: (c, d) MW-NH_2_ or (e, f) MW-COOH. (a) *IL-6* and (b)* TGF-*β*1 *low labelling detected in control rats at all lung districts (e.g., bronchiolar, stromal, and alveolar levels). (c, e) Strongly *IL-6*-immunoreactive bronchiolar areas and collapsed alveoli, showing several markedly immunopositive cells (*arrows*), together with the presence of widespread black particulate. (d, f) Strong *TGF-*β*1 *immunoreactivity at stromal level *(arrows)* of some collapsed alveolar zones, with evident immunopositive cells *(arrows). Objective magnification: *40x.

In agreement with previous literature data reporting toxic pulmonary effects including inflammation (characterized by an increase in alveolar cell number and in cytokines, e.g., TNF-alpha and IL-1) and oxidative stress [24, 25, 44, 45, 55], our experimental results showed an extensive spreading in the bronchiolar, alveolar, and stromal cells of pulmonary IL-6 and TGF*β*1, evidencing the cellular inflammatory reaction to the CNTs instillation (Table 2 and Figure 7), while, differently, evident changes for collagen were not detected.

The persistence of inflammation, still observable 16 days after instillation, could be explained as the consequence of failure to eliminate the inciting agent (i.e., different MWCNTs), as demonstrated by the durability of fibrocarbonaceous nanomaterial internalized by macrophages. Differently, the lack of a marked collagen accumulation could be attributable to a physiological tissue repair in which the tissue remodelling occurred moderately.

These research studies on CNTs clearly indicated that certain negative properties that are typical of classic CNTs such as cytotoxicity, poor blood compatibility, inflammogenic effects, and target-organ toxicity are maintained to some extent in functionalized CNTs, supporting the view that overcoming the safety problems remains a considerable challenge for these nanomaterials.

## 4. Conclusions 

In summary, our first *in vitro *step investigation reported conflicting results on the cytotoxic effects of *pristine* and differently functionalized MWCNTs; specifically all the tested CNTs exhibited mild to moderate cytotoxicity when tested using MTT assay, with changes occurring not dose-dependently already at very low CNT concentrations. By contrast, the calcein/PI test showed that cell viability was unaffected by all tested CNTs, even at the higher concentrations and for longer exposure time. These diverging results highlighted some limitations intrinsical to classical *in vitro* cytotoxicity tests, applied to the study of the ENMs, due to the peculiar physicochemical characteristics of these new nanomaterials, fully supporting the notion [56, 57] that a number of issues remain to be resolved before the exclusive use of *in vitro* studies can be accepted in the safety evaluation of nanomaterials, calling for research approaches complementary to the *in vitro* studies aimed at understanding effects at all physiological levels and predicting human health hazards.

Strategies for the safety assessment of ENMs and agglomerates are under discussion and development in several national and international projects. It has been suggested [58] to develop a tiered strategy of tests starting with an assessment of cell viability providing a first-stage attempt at screening particles, allowing their benchmarking, before choosing specific particles for further testing, perhaps *in vivo*.

Since investigations in laboratory animals may give essential insight, the *in vivo* study, being the subsequent second essential stage, has provided fundamental information, contributing to clarifying the CNT toxicity mechanisms and to understanding the different CNT toxicity targets, at different pulmonary cytochemical levels, in order to improve the overall understanding of the possible adverse outcomes resulting from CNT exposure.

In particular, our *in vivo* data demonstrated that intratracheal instilled MWCNTs can induce lung toxicity associated with inflammation, even in absence of fibrosis, irrespective of nanotubes functionalization. This latter finding suggests caution before considering chemical functionalization as a broad way to improve biocompatibility and safety characteristics of CNTs, being even more important for CNTs that are proposed for diagnostic or therapeutic applications.

These overall experimental results further support the use of a multitiered strategy for the safety assessment of the human health impact of novel nanotechnology-based products.

## Figures and Tables

**Table 1:** Physicochemical properties of nanoparticulate materials.

	MWCNTs	MW-COOH	MW-NH_2_	CB	SiO_2_
Primary particle size range (nm)	500–2000	100–300	100–300	10–20	7
Aggregate size range (nm)	≤2000*	300–1000*	300–1000*	100–1000	150–200
Specific surface area BET (m^2^/g)	60	~100	~100	~240	~200
Zeta potential (mV) in deionised H_2_O	−44	−50	−48	−22	−30

*Determined by NC-AFM on samples deposited on high resolution mica sheets.

**Table 2:** Localization and expression of immunolabeling for IL-6, TGF-*β*1 and Collagen (type I) on a semiquantitative evaluation.

	IL-6	TGF-*β*1	Collagen-I
Control			
Bronchiolar cells	±	−	+
Alveolar cells	±	−	±
Stromal cells	±	±	+
*Pristine* MW			
Bronchiolar cells	++±	+±	+±
Alveolar cells	+±	+	+
Stromal cells	+±	+++	+±
MW-NH_2_			
Bronchiolar cells	++++	++	+±
Alveolar cells	++	+	+±
Stromal cells	++±	++++	++
MW-COOH			
Bronchiolar cells	++±	++	+±
Alveolar cells	+±	+	+±
Stromal cells	+±	+++±	++

*P* value			
Bronchiolar cells	<0.05	<0.05	ns
Alveolar cells	<0.05	<0.05	ns
Stromal cells	<0.05	<0.05	ns

Degree of staining intensity: from undetectable (−) to strong (++++).

*P* values calculated by Kruskal-Wallis test.

ns: not statistically significant.
